# What Is Excitation/Inhibition and How Is It Regulated? A Case of the Elephant and the Wisemen

**DOI:** 10.1177/1179069519859371

**Published:** 2019-06-23

**Authors:** Hai-yan He, Hollis T Cline

**Affiliations:** The Scripps Research Institute, La Jolla, CA, USA

**Keywords:** Excitation/inhibition balance, spontaneous synaptic current, sensory-evoked synaptic current, intrinsic excitability, circuit dysfunction

## Abstract

The balance between excitation and inhibition in neuronal circuits has drawn more and more attention in recent years, due to its proposed multifaceted functions in the normal neural circuit as well as its potential roles in the etiology of many neurological disorders. Here, we discuss the importance of clearly defining excitation/inhibition by experimental measurements and the implications of some recent studies to our understanding of the regulation of excitation/inhibition at the neuronal level.

**Comment on:** He HY, Shen W, Zheng L, Guo X, Cline HT. Excitatory synaptic dysfunction cell-autonomously decreases inhibitory inputs and disrupts structural and functional plasticity. *Nat Commun*. 2018;9:2893. doi: 10.1038/s41467-018-05125-4. PubMed PMID: 30042473; PubMed Central PMCID: PMC6057951. https://www.ncbi.nlm.nih.gov/pubmed/PMC6057951

Neurons and circuits coordinate their excitatory and inhibitory inputs to establish and maintain a constant excitation/inhibition (E/I) ratio that is thought to be essential for circuit function and stability. Theoretical modeling demonstrated that when inhibition tightly matches excitation and tracks it on milliseconds time scale in the neural network, it provides great advantage to the precision and efficiency of neuronal coding mechanisms.^1^ Experimental evidence also supports the idea that balanced excitation and inhibition within neural circuits facilitates their function,^2–4^ and that failure to maintain E/I balance underlies circuit dysfunction seen in many neurological diseases. Nevertheless, how a balance of excitatory to inhibitory inputs is established and subsequently maintained remains a subject of debate. Recent studies suggest that multiple cellular mechanisms contribute to the regulation of E/I balance.

## First, What Is E/I? and How Should It Be Measured?

In the literature, E/I is often referred to in a very broad sense. In most neurons studied, certainly the most intensively studied cortical and hippocampal neurons, inhibitory inputs that one cell receives usually originate from a variety of inhibitory neurons, which in turn receive highly diverse inputs.^5^ Furthermore, different subtypes of inhibitory neurons can assume a different valence of plastic changes in response to experience.^6,7^ The I (inhibition) in E/I can mean very different things. Sometimes it refers to a subset of the inhibitory inputs (as examined in many studies using transgenic mouse line that focuses on subtypes of inhibitory neurons). Sometimes it refers to ALL inhibitory inputs a cell receives (as in cases when spontaneous or sensory-evoked synaptic activity was examined). The temporal window over which the excitatory and inhibitory synaptic currents are included in the E/I measurement can also vary across studies. Sometimes it is summed over a relatively wide temporal window (hundreds of milliseconds), such as shown in visual cortical neurons.^8^ Sometimes it is confined to a very narrow temporal window with delays on several milliseconds scale, as is the case in auditory and somatosensory cortical neurons.^9,10^ Another question concerns the method used to measure E/I. Is measurement of spontaneous synaptic events (miniature excitatory postsynaptic current [mEPSC] and miniature inhibitory postsynaptic current [mIPSC]) sufficient to reflect E/I or should evoked events be measured? Although E/I balance recorded by spontaneous synaptic currents may largely agree with E/I measured by evoked synaptic activity,^11^ measuring E/I by recording compound excitatory and inhibitory synaptic currents in vivo evoked by sensory stimulation may be more physiologically relevant, since this reflects the outcome of the coordinated network activity. On the other hand, recording spontaneous inhibitory and excitatory synaptic currents, and similarly, quantifying excitatory and inhibitory synaptic markers post hoc, reveals global information about average inputs to the neuron, independent of which inputs are activated by a particular stimulus. Likewise, optogenetic or electric stimulation in brain slices would potentially evoke all synaptic inputs within the pathway but not necessarily reflect the full complement of synaptic inputs evoked by sensory stimulation.

This is an important point because the measurement, and therefore the definition of E/I, may pertain to its function. Multiple functions of E/I have been proposed. For example, the temporally tightly matched E and I circuits described in sensory cortices can only be measured by sensory-evoked synaptic responses and may be essential for efficient coding of sensory information,^1^ while the balance of E and I at the level of single neurons and circuits in a more general sense may safeguard circuit stability.^12,13^ Whether the same or different E and I circuits serve these different functions is unclear and this ambiguity may call for more cautious interpretations of experimental results in E/I studies.

## Second, How Is E/I Established and Maintained?

Neurons (and circuits) maintain E/I balance in response to fluctuations of input activity^7,14^; however, knowledge of how E/I balance is established and what triggers adjustments of E or I to maintain the balance of E/I remains scarce. Different methods have been used to perturb inputs/outputs of neurons in vivo and in vitro, but not all manipulations triggered corresponding adjustment of E and I that resulted in unchanged E/I ratio, as measured by E and I synaptic inputs.

One way to perturb the neuronal inputs is to interfere with synaptic inputs. To test whether E/I balance can be maintained after perturbing excitatory synaptic inputs, we interfered with AMPAR (α-amino-3-hydroxy-5-methyl-4-isoxazolepropionic acid receptor) trafficking into glutamatergic synapses and evaluated the effect of this manipulation on inhibitory synapses using both electrophysiology and imaging methods in the visual system of Xenopus tadpoles. This experimental system allows time-lapse morphological studies to evaluate the effects of manipulating synaptic inputs on individual neurons,^11^ and in vivo electrophysiological recordings of spontaneous and visually evoked responses, as well as visually evoked behavior to assess circuit-level effects of manipulating E/I. At the single cell level, interfering with excitatory synaptic transmission decreased mEPSCs, as expected. Surprisingly, this also led to a cell autonomous decrease in mIPSCs, as well as a decrease in the density of inhibitory synaptic puncta. Furthermore, both excitatory and inhibitory components of visually evoked responses were proportionally decreased in the affected optic tectal neurons, suggesting that decreasing excitatory synaptic input induced a cell-autonomous down-regulation of inhibitory synaptic input, so that the E/I balance was maintained. This was in stark contrast to our previous observation that decreasing inhibitory inputs to tectal cells, by expressing a peptide that interferes with GABA_A_R (gamma-aminobutyric acid type A receptor) trafficking into synapses, does not induce a corresponding decrease in excitatory inputs.^15^ This indicates that maintaining E/I is not an automatic cellular response to any perturbance of either inhibitory or excitatory inputs to the cell but is specifically induced by changes in the excitatory inputs. This “leading” role of excitation over the regulation of E/I is also consistent with the observations that the maturation of inhibition trails excitation in sensory cortices during development.^16–18^

An alternate strategy to probe mechanisms regulating E/I has been to decrease excitability, for instance, with potassium channel expression. Expressing Kir2.1 in a subset of layer 2/3 pyramidal neurons reduced inhibitory inputs from PV+ neurons without affecting excitatory synaptic inputs.^19^ Furthermore, changes in synaptic inputs from the same presynaptic PV inhibitory neurons were specific to the Kir2.1-expressing postsynaptic cells, suggesting cell-autonomous regulation of the PV input by a retrograde signal from the postsynaptic neuron. In other studies, decreasing postsynaptic spiking in cultured neurons induced a homeostatic increase in excitatory synaptic inputs but failed to affect inhibitory synaptic inputs.^20,21^ It is possible that modulating intrinsic excitability is yet another mechanism that neurons employ to maintain its input/output stability, in addition to E/I. In all the above cases, when intrinsic excitability was perturbed, either excitatory or inhibitory synaptic inputs changed accordingly, presumably to maintain neuronal output, but rarely did E and I changed simultaneously, which resulted in disrupted E/I as measured by synaptic inputs. Interestingly, manipulating CaMKIV (calcium/calmodulin-dependent protein kinase type IV) activity in individual pyramidal neurons triggered cell-autonomous changes in both excitatory synaptic inputs and intrinsic excitability, but failed to induce any change in the inhibitory synaptic inputs,^22^ also suggesting a dissociation of the co-regulation of E and I synaptic inputs when intrinsic excitability was changed.

These studies highlight the strikingly different effects of modifying excitability and excitatory synaptic inputs with respect to regulation of E/I and importantly indicate that E/I is not maintained through a single consensus mechanism. Both intrinsic excitability and excitatory synaptic inputs affect neuronal output (spiking), but through different mechanisms. Intrinsic excitability affects the input-output function, whereas excitatory synaptic input mostly affects the inputs, and both may affect the active membrane properties in local dendrites. Reduced excitatory input is not equivalent to reduced excitability of the neurons. Reduction in excitability suppresses spiking activity of the neuron but does not prevent postsynaptic depolarization evoked by excitatory synaptic inputs, which allows local Ca influx and can trigger downstream signaling pathways.^23^ As pointed out above, although excitatory inputs and intrinsic excitability have been reported to be co-regulated under certain circumstances,^20–22^ there are also cases when they are separately regulated.^19^ It is possible that multiple mechanisms are involved in the maintenance of input/output stability. Whether changes in excitatory synaptic inputs and changes in intrinsic excitability should be treated similarly in terms of E/I regulation remains a question. Data from our studies provide strong in vivo evidence that inhibitory synaptic inputs are regulated cell-autonomously in response to a direct disruption of excitatory synaptic inputs, which maintains the E/I balance similar to that in the control neurons. It remains to be seen whether intrinsic excitability was also changed in this scenario.

## Third, Is Control of E/I Balance Cell-Type Specific? Do Inhibitory Neurons Regulate Their E/I Balance?

To this day, most studies on E/I focus on excitatory neurons, which is not surprising, as they execute neuronal coding. Nonetheless, since inhibitory neurons also receive both excitatory and inhibitory inputs, it is important to determine whether they also regulate E/I balance. Studies in hippocampus indicate that both the excitatory and the inhibitory neurons can adjust their intrinsic excitability in response to changes in activity,^24^ hinting that they may also regulate E/I. We investigated the effect of GluA-CTP expression on E/I in inhibitory neurons. Our study demonstrated that in inhibitory neurons, at both synaptic and cellular levels, cell-autonomous decreases in inhibitory inputs were induced by decreased excitatory synaptic transmission and subsequently maintained E/I, similar to that observed in excitatory neurons.^11^ This indicates that excitatory and inhibitory neurons both maintain E/I balance in response to changes in excitatory synaptic input activity. However, the mechanisms that excitatory and inhibitory neurons utilize for the maintenance of E/I balance may differ. One clue is that Npas4, an immediately early gene induced by neuronal activity, is known to recruit somatic inhibition onto excitatory neurons in response to increased neuronal activity. Knocking out Npas4 in somatostatin (SST)-positive inhibitory neurons significantly decreased excitatory synaptic inputs but not inhibitory synaptic inputs,^25^ suggesting that the excitatory and inhibitory neurons may employ distinct activity-dependent programs regulating excitatory and inhibitory inputs.

## Future Perspectives

Excitation/inhibition has been studied extensively regarding normal neuronal function and stability, and disruption of E/I has been implicated in many neurological diseases. Yet, the exact meaning of E/I has become rather ambiguous and varies greatly with context. To dissect the circuits underlying the different functions proposed for E/I, as well as understand the molecular mechanisms underlying the maintenance of E/I, it is critical to specify the way that E/I is measured. In terms of the functional relevance of E/I balance, multiple aspects of E/I should be taken into consideration. For instance, although there is abundant evidence suggesting that E/I balance is necessary for the normal function of neural circuits, few studies have explicitly tested whether E/I is sufficient for circuit function. Our behavioral data suggest that is not the case.^11^ Despite the relatively normal E/I ratio assessed as synaptic currents (both visually evoked and spontaneous), the decreased excitatory and inhibitory synaptic inputs in tectal neurons following AMPAR trafficking interference significantly compromised their receptive field properties and ramified extensively throughout the visuomotor circuit, interfering with visual experience-dependent dendritic arbor plasticity, visual receptive fields, and visuomotor behavior. These defects are likely due to the disruption of the fine temporal balance (match) between the visually evoked excitatory and inhibitory inputs after interfering with AMPAR trafficking into excitatory synapses,^11^ suggesting that temporal features also need to be considered when E/I balance is concerned.
